# *Neopestalotiopsis* spp., an invasive fungal pathogen, is a major threat to strawberry production: the current status, challenges, and future directions

**DOI:** 10.3389/fpls.2026.1725321

**Published:** 2026-01-28

**Authors:** Susmita Gaire, Norman Muzhinji, Frank J. Louws, Tika B. Adhikari

**Affiliations:** 1 Department of Entomology and Plant Pathology, North Carolina State University, Raleigh, NC, United States; 2 University of the Free State, Department of Plant Sciences, Plant Pathology Division, Bloemfontein, South Africa; 3 Department of Horticultural Science, North Carolina State University, Raleigh, NC, United States

**Keywords:** *Fragaria* × *ananassa*, fungal disease, fungal invasive pathogen, neopestalotiopsis, strawberry cultivars, virulence, genomic tools, pathogen detection

## Abstract

Pestalotioid fungi have traditionally been regarded as secondary or opportunistic pathogens of strawberries, which has led to limited research attention. However, recent outbreaks of *Neopestalotiopsis* have demonstrated its potential to act as a primary pathogen, posing a significant threat to strawberry production worldwide. Current management strategies primarily involve propagation of pathogen-free plants, cultural practices such as field sanitation, crop rotation, and the removal of infected plants, supplemented by the application of biocontrol agents and fungicides. Advances in molecular diagnostic tools have improved early detection and monitoring of *Neopestalotiopsis* spp. Furthermore, initial efforts have begun to identify sources of genetic resistance in strawberry, thereby supporting future breeding programs. Despite these advancements, a considerable gap remains in our understanding of the host’s defense mechanisms, the pathogen’s infection strategies, the dynamics of their interactions, and the pathogen’s ecology. The taxonomy’s complexity and the variability in virulence among its isolates further complicate diagnosis and control efforts. Addressing these challenges is crucial to developing sustainable, integrated disease management strategies and advancing resistance breeding, thereby ensuring the long-term productivity and resilience of the strawberry industry. This review consolidates the current understanding of *Neopestalotiopsis* spp., evaluates the available diagnostic tools and management strategies, discusses recent progress in genetics and genomics for breeding resistance to this pathogen, and identifies areas for further research.

## Introduction

**Figure 1:**
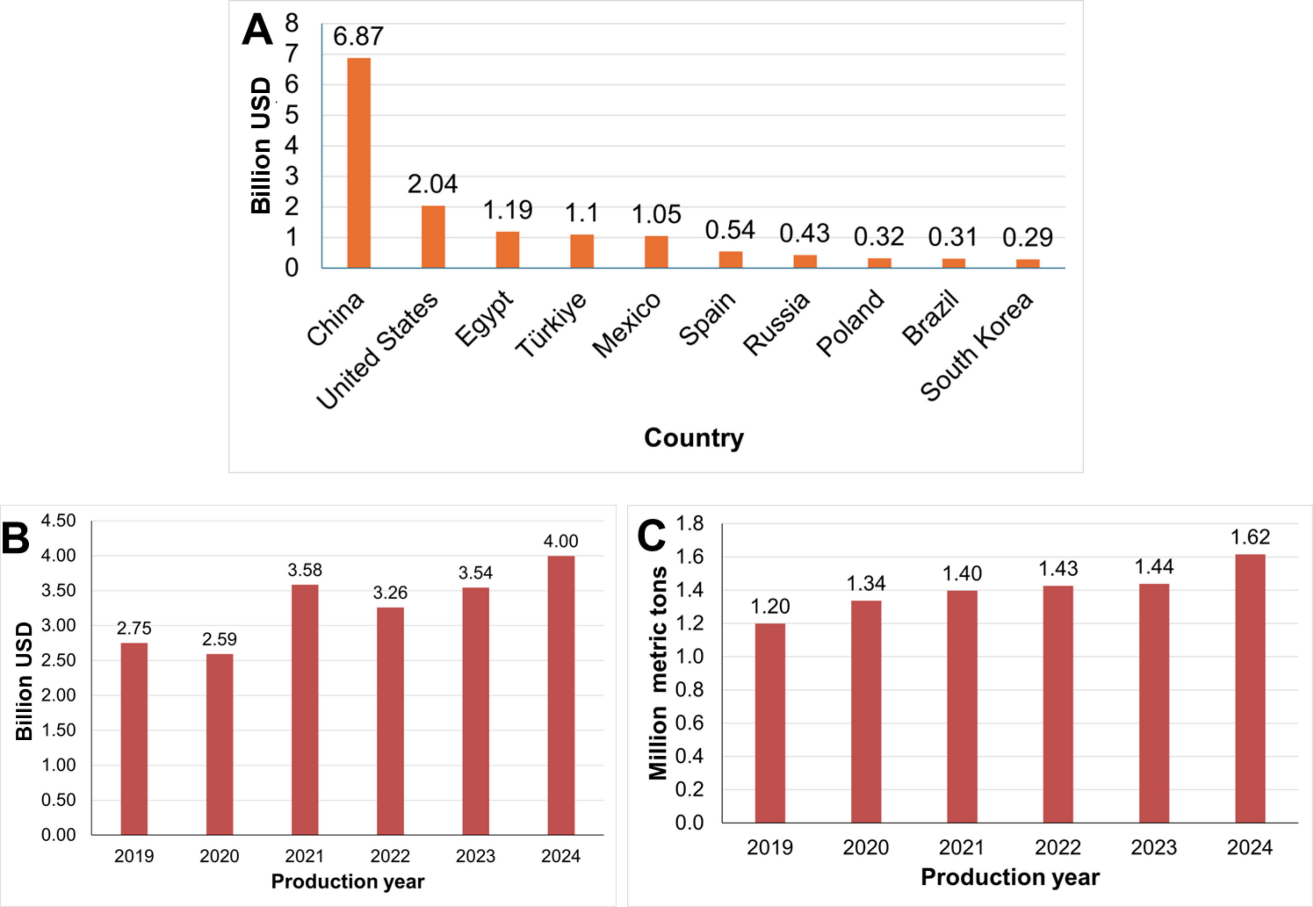
Economic importance of strawberries showing **(A)** the value of strawberry production in billion U.S. dollars (USD) in the top 10 producing countries in the year 2023; **(B)** the value of strawberry production in billion USD in the United States from 2019 to 2024, and **(C)** the production volume of strawberries in million metric tons in the United States from 2019 to 2024.

Cultivated strawberry (*Fragaria × ananassa*) is one of the world’s most important fruit crops, valued for its high nutritional content, including vitamin C, antioxidants, and dietary fiber, as well as its significant economic contribution. Globally, strawberries represent a multibillion-dollar industry, with production exceeding 9, 7 million metric tons and a market value of over $15.9 billion in 2023 (FAOSTAT, 2025). Major producing countries include China, the United States, Egypt, Turkey, and Mexico (FAOSTAT, 2025; **Figures 1A, B**). In the U.S., the fresh strawberry industry achieved an estimated $4 billion in 2024, with 1.6 million metric tons of production, an increase of over 12% in both market size and yield compared to the previous year (**Figure 1C**; USDA-NASS, 2025).

Despite this economic success, strawberry production is highly vulnerable to abiotic and biotic stresses. Abiotic factors such as temperature extremes (Gulen and Eris, 2003, 2004; Roussos et al., 2020; Shokaeva, 2008), storage temperature fluctuations (Xing et al., 2025), suboptimal soil conditions (Yao et al., 2015), and drought (Ünal and Okatan, 2023; Zahedi et al., 2023), among others, can severely affect qualitative and quantitative yield. In addition to these challenges, strawberries are susceptible to a wide range of pathogens that affect fruit, shoots, crowns, and roots (Maas, 1998), thereby severely impacting productivity. Recently, the invasive fungal genus *Neopestalotiopsis* has emerged as a major threat to strawberry cultivation worldwide, particularly in warm and humid environments (Ávila-Hernández et al., 2025; Baggio et al., 2021; Dardani et al., 2025; Guan et al., 2023; McNally et al., 2023; Uriel et al., 2025). A notable outbreak of leaf spot and fruit rot in Florida in 2017 marked the beginning of widespread *Neopestalotiopsis* infections across the southeastern U.S., particularly in fruiting fields and propagation nurseries (Baggio et al., 2019, 2021). During the 2018–2019 season, Florida growers reported losses of 40–50% in established fields (Baggio et al., 2019), underscoring the significant economic impact of this rapidly spreading pathogen.

*Neopestalotiopsis* outbreaks are closely linked to the use of infected nursery transplants, prolonged rainfall, and high humidity (Baggio et al., 2021; Rebollar-Alviter et al., 2020; Zuniga et al., 2024). The pathogen’s aggressiveness and persistence in plant debris, soil, and other inoculum reservoirs ensure season-to-season survival and rapid re-emergence under conducive conditions, making management particularly challenging (Baggio et al., 2021; Zuniga et al., 2024). To date, disease management strategies have relied primarily on an integrated approach combining cultural and chemical methods. These include planting pathogen-free plants, if available, field sanitation practices, crop rotation to reduce inoculum build-up, removal of infected plants, application of biocontrol agents, and fungicide treatments (Amrutha and Vijayaraghavan, 2018; Baggio et al., 2019, 2023; Goura et al., 2025; Zuniga et al., 2024). However, fungicide use is constrained by the limited efficacy of registered products against this pathogen complex, regulatory constraints on application frequency, environmental sustainability concerns, and the emergence of fungal resistance in pathogen populations.

Molecular diagnostic techniques have emerged as valuable tools for the early and accurate detection of *Neopestalotiopsis* spp., enhancing nursery detection systems and field-level surveillance (Kaur et al., 2023; Rebello et al., 2023). Despite these advances, their adoption in commercial production systems remains limited, forcing growers to rely on conventional diagnostic methods that are subjective due to taxonomic confusion and homonyms arising from significant morphological overlap among *Neopestalotiopsis* species (Maharachchikumbura et al., 2014). Bridging the gap between research advancement and field implementation is critical for the sustainable management of *Neopestalotiopsis* outbreaks in strawberry production worldwide.

Taxonomic complexity and limited genomic resources of *Neopestalotiopsis* hinder accurate identification and understanding of pathogen diversity, key requirements for developing targeted management strategies (Kaur et al., 2023; Maharachchikumbura et al., 2014; Rebello et al., 2023). Aggressive *Neopestalotiopsis* isolates have been detected in multiple U.S. states, yet most commercial strawberry cultivars lack known genetic resistance (Baggio et al., 2019, 2021; Guan et al., 2023). Breeding efforts are further constrained by the absence of well-characterized resistance sources in both cultivated and wild germplasm, with the cultivar ‘Yasmin’ being the only exotic cultivar exhibiting promising sources of resistance (Alam et al., 2024). In addition, the allo-octoploid nature of cultivated strawberry species (2n = 8× = 56) complicates genetic research and breeding initiatives (Edger et al., 2019). Given the increased prevalence of *Neopestalotiopsis*, its significant economic impact, and the limited effectiveness of available control measures, there is a critical need for comprehensive, multidisciplinary research efforts. These efforts should combine pathogen biology, diagnostics, epidemiology, and integrated management strategies, including advances in breeding, to address these issues.

### Review aim

This study aims to review the current state, identify challenges, and outline future directions of an emerging disease caused by the invasive fungal pathogen *Neopestalotiopsis* in strawberry production systems. This review primarily focuses on the strawberry as the most economically significant host while also expanding the discussion to include symptoms and infection process in other host crops where applicable.

### Review objectives

1. To review the current conventional and molecular technologies or tools used to investigate the biology of *Neopestalotiopsis* spp. and manage the associated disease.

2. To evaluate the current challenges faced in managing the disease.

3. To explore future directions and innovative approaches that can guide the study of the pathogen and improve disease management strategies.

## Taxonomy and morphological characteristics of *Neopestalotiopsis*

**Figure 2:**
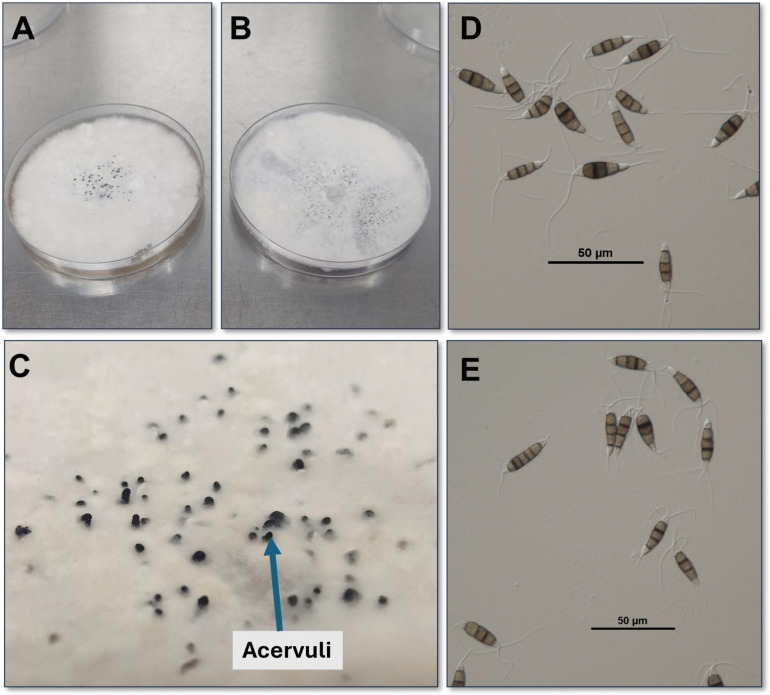
Fungal culture on potato dextrose agar (PDA) medium, showing acervuli and conidia of *Neopestalotiopsis* from North Carolina. **(A)** PDA plates with NC4 isolates (*Neopestalotiopsis rosae*), **(B)** PDA plates with NC20 isolates (*Neopestalotiopsis* spp.), **(C)** Acervuli **(D)** Conidia of NC4 isolates (*Neopestalotiopsis rosae*), **(E)** Conidia of NC20 isolates (*Neopestalotiopsis* spp.).

*Neopestalotiopsis* was first identified as a distinct genus by Maharachchikumbura et al. (2014), following a comprehensive reclassification of *Pestalotiopsis*. This revision was based on phylogenetic analyses that integrated molecular data from the internal transcribed spacer (ITS), partial β-tubulin (TUB), and translation elongation factor 1-alpha (TEF) gene regions, along with conidial morphology. As a result, the genus *Pestalotiopsis* was divided into three distinct genera: *Pestalotiopsis*, *Neopestalotiopsis*, and *Pseudopestalotiopsis* (Maharachchikumbura et al., 2014). Morphologically, all three genera produce conidia composed of five cells: three median cells and two terminal cells (one basal and one apical) (**Figures 2A–E**). *Neopestalotiopsis* can be distinguished by its variegated (versicolorous) median cells, in contrast to the uniformly colored (concolorous) median cells of *Pestalotiopsis* and *Pseudopestalotiopsis* (Maharachchikumbura et al., 2014). Specifically, the median cells of *Neopestalotiopsis* conidia are light brown, honey-brown, or brown, whereas the apical and basal cells are hyaline (Baggio et al., 2021; Maharachchikumbura et al., 2014). *Neopestalotiopsis conidia* are typically ellipsoid to fusiform in shape. They are characterized by an apical cell that bears 2 to 4 branched or unbranched tubular appendages and a basal cell with a single unbranched appendage (Baggio et al., 2021; Maharachchikumbura et al., 2014; Rebollar-Alviter et al., 2020). These appendages extend as tubular structures from the conidium body, maintaining protoplasmic continuity (Maharachchikumbura et al., 2014).

Colony morphology and mycelial growth vary significantly among *Neopestalotiopsis* spp. Maharachchikumbura et al. (2014) described these colonies as being white, pale honey, or pale yellow, with sparse to dense aerial mycelium on both the upper and lower surfaces. These colonies often feature black, concentric, or gregarious conidiomata. Additionally, other studies have noted variations in mycelial pigmentation and texture across species (Baggio et al., 2021). For instance, the newly identified *Neopestalotiopsis* spp. and *Neopestalotiopsis rosae*, collected in Florida, exhibited distinct morphologies: the former had white to pale-yellow surfaces. At the same time, the latter showed a pale luteous to orange lower surface (Baggio et al., 2021). Both *N. rosae* and the newly described *Neopestalotiopsis* spp. produced white, circular, cottony growth on their upper surfaces (Baggio et al., 2021).

The genus *Neopestalotiopsis* belongs to the family *Sporocadaceae* (Razaghi et al., 2024) and comprises 131 species, currently listed in Index Fungorum (https://www.indexfungorum.org/names/Names.asp). Members of this genus are predominantly characterized by an asexual mode of reproduction, in which conidia are formed within conidiomata, which serve as the primary source of inoculum. Additionally, two teleomorph genera, *Neobroomella* (Kirk et al., 2008) and *Pestalosphaeria* (Barr, 1975), are associated with the broader *Pestalotiopsis* complex (Maharachchikumbura et al., 2011). Among the 12 sexually reproducing *Pestalosphaeria* species, 11 are linked to *Pestalotiopsis*, except for *Pestalosphaeria maculiformans*, which is the sexual morph of *Pestalotiopsis maculiformans* (Maharachchikumbura et al., 2014, 2011). Recent taxonomic revisions have reclassified *Pestalotiopsis maculiformans* under *Neopestalotiopsis* due to the presence of versicolored median cells (Maharachchikumbura et al., 2014). Consequently, *Pestalosphaeria maculiformans* is recognized as the only known teleomorphic state within the genus *Neopestalotiopsis*.

## *Neopestalotiopsis:* global distribution and host range

**Figure 3:**
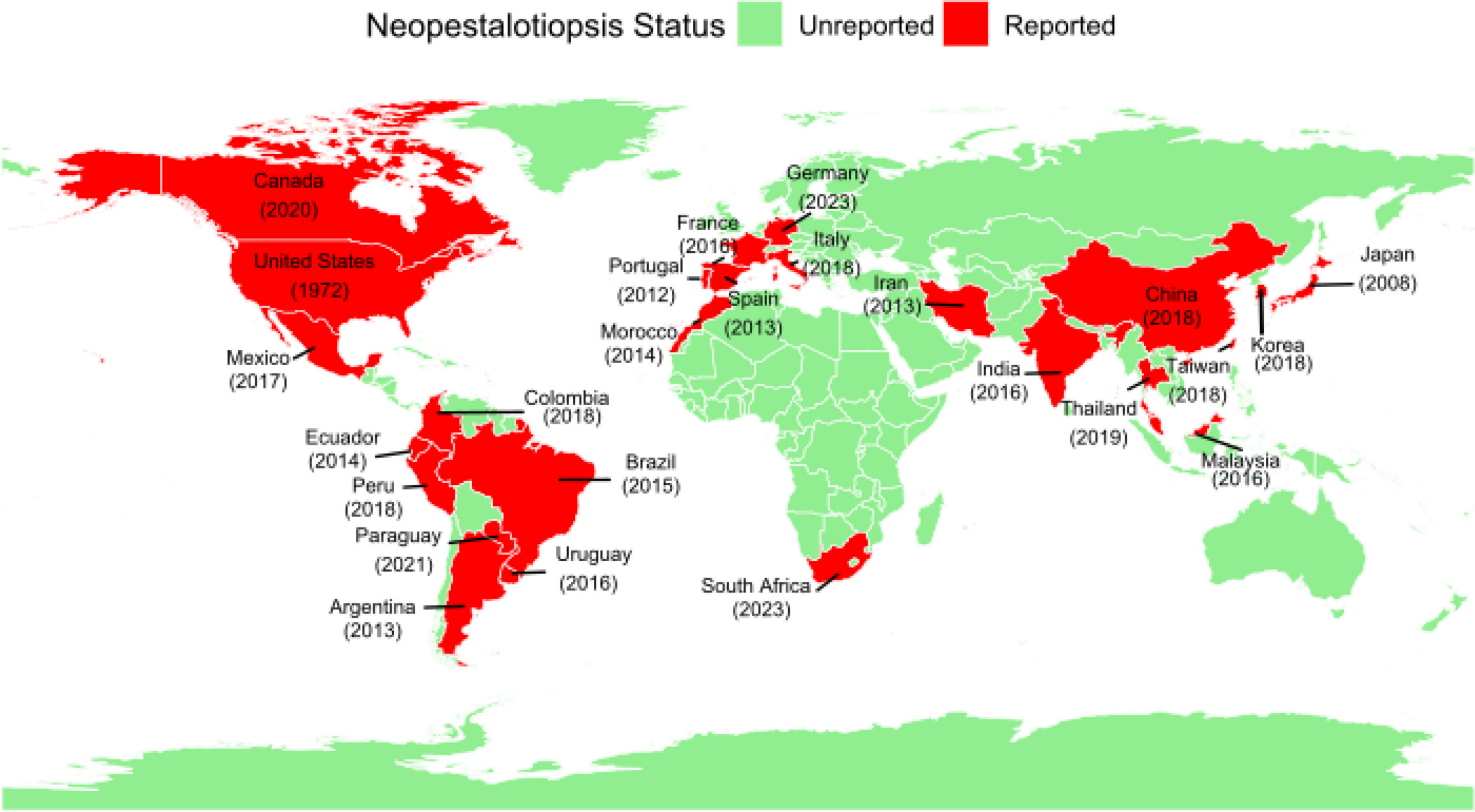
Global distribution of *Neopestalotiopsis* spp. across hosts, with red indicating countries where *Neopestalotiopsis* spp. has been reported. Numbers in parentheses indicate the year of first reporting. The figure is based on the data in **Table 1**.

*Neopestalotiopsis* is a globally distributed fungal genus that is commonly found in tropical, subtropical, and temperate regions (**Figure 3**, **Table 1**).

**Table 1:** *Neopestalotiopsis* spp. are listed with their global distribution and host range, including the countries and years reported, host crops, symptoms, and the *Neopestalotiopsis* spp. isolated.

Location	Year of sample collection	Crop	Symptom isolated	Species	Reference
Argentina	2013	Strawberry	Root and crown rot	*N. clavispora*	(Obregón et al., 2018)
Australia	2012/13	Macadamia	Dry flower	*Neopestalotiopsis macadamiae* sp. *nov.*	(Akinsanmi et al., 2017)
Australia	2012/13	Macadamia	Dry flower	*Neopestalotiopsis* spp.	(Prasannath et al., 2021)
Brazil	2015	Macadamia	Leaf spot	*N. clavispora*	(Santos et al., 2019)
Brazil	2018	Coconut	Leaf spot	*N. foedans*	(Barbosa et al., 2023)
Brazil	2020	Eucalyptus	Stem rot	*Neopestalotiopsis* spp.	(Santos et al., 2020)
Brazil	2014 -2016	*Arecaceae*	Leaf spot	*Neopestalotiopsis* spp.	(Guterres et al., 2023)
Canada	2020	Strawberry	Leaf spot	*Neopestalotiopsis* spp.	(McNally et al., 2023)
Canada	2021 - 2022	Ligonberry	Leaf spot and stem dieback	*N. rosae* and *N. zimbabwana*	(Novinscak et al., 2025)
China	2018	Macadamia	Leaf spot	*N. clavispora*	(Qiu et al., 2020)
China	2018	Rosa chinensis	Stem canker	*Neopestalotiopsis rosicola* sp. *nov.*	(Jiang et al., 2018)
China	2018	*Camellia sinensis*	Gray blight	*N. piceana*	(Wang et al., 2023b)
China	2018	*Paphiopedilum micranthum* (Silver slipper orchid)	Leaf spot	*N. saprophytica*	(Qin et al., 2020)
China	2018	Strawberry	Root rot	*N. rosae*	(Sun et al., 2021)
China	2019	*Camellia chrysantha*	Leaf spot	*N.* spp.	(Zhao et al., 2020)
China	2019	*Taxus media*	Branch blight	*N. clavispora*	(Li et al., 2022)
China	2020	Strawberry	Calyx and receptacle blight	*N. clavispora*	(Shi et al., 2022)
China	2020	Rubber Tree	Leaves lesion	*N. aotearoa*	(Li et al., 2021)
China	2020	Banana (*Musa acuminata*)	Leaf spot	*N. clavispora*	(Qi et al., 2023)
China	2021	Apple	Leaf spot	*Neopestalotiopsis clavispora*	(Shi et al., 2024)
China	2021	Guava	Guava scab	*N. clavispora*	(Lin et al., 2024)
China	2021	Strawberry	Leaf spot	*N. fragariae*	(Prematunga et al., 2022)
China	2023	Rhododendron	Leaf spot	*N. terricola*	(Li et al., 2025)
Colombia	2018	Guava	Guava scab	*Neopestalotiopsis* spp.	(Solarte et al., 2018)
Ecuador	2014	Strawberry	Root and crown rot	*N. mesopotamica*	(Hidrobo-Chavez et al., 2022; Intriago-Reyna et al., 2021)
France	2016	Grapevine	Trunk disease	*Neopestalotiopsis* spp.	(Maharachchikumbura et al., 2016)
Germany	2023	Strawberry	Leaf blight and fruit rot	*N. rosae*	(Schierling et al., 2024)
India	2023	Strawberry	Leaf spot and fruit rot	*N. javaensis*	(Rakhonde et al., 2025; Mahapatra et al., 2018)
Iran	2013	Strawberry	Fruit rot	*N. iranensis* sp. *nov.*, and *N. mesopotamica*	(Ayoubi and Soleimani, 2016)
Italy	2018	Strawberry	Root and crown rot	*N. clavispora*	(Gilardi et al., 2019)
Italy	2020	Avocado	Stem lesion and dieback	*Neopestalotiopsis siciliana* sp. nov. and *N. rosae*	(Fiorenza et al., 2022)
Italy	2023/2024	Strawberry	Crown rot	*Neopestalotiopsis* species	(Dardani et al., 2025)
Japan (Hakone, Kanagawa)	2016	Japanese andromeda	Leaf blight	*Neopestalotiopsis* spp.	(Nozawa et al., 2019)
Japan (Tokyo)	2008	Japanese andromeda	Leaf blight	*Neopestalotiopsis* spp.	(Nozawa et al., 2019)
Korea	2018	Blueberry	Twig dieback	*N. clavispora*	(Lee et al., 2019)
Malaysia	2016	Oil Palm (*Elaeis guineensis*)	Leaf spot	*N. saprophytica*	(Ismail et al., 2017)
Malaysia	2019	Tea	Grey leaf blight	*N. clavispora*	(Shahriar et al., 2022)
Mexico	2017	Mango	Grey leaf spot	*Neopestalotiopsis* spp.	(Gerardo-Lugo et al., 2020)
Mexico	2017	Strawberry	Leaf spot, root, and crown rot	*N. rosae*	(Rebollar-Alviter et al., 2020)
Morocco	2014	Strawberry	Leaf spot and fruit rot	*N. rosae*	(Mouden et al., 2014)
Morocco	2019/20	Grapevine	Trunk diseases	*Neopestalotiopsis vitis*	(Kenfaoui et al., 2024)
Paraguay	2021	Strawberry	Leaf spot and crown rot	*N. rosae*	(Fernández-Ozuna et al., 2023)
Peru	2018	Blueberry	Stem blight and die back	*Neopestalotiopsis* spp.	(Rodríguez-Gálvez et al., 2020)
Portugal	2012	*Eucalyptus* spp.	Leaf necrosis, stem girdling, and cutting dieback	*N. eucalyptorum, N. hispanica, N. iberica, N. longiappendiculata* and *N. lusitanica.*	(Diogo et al., 2021)
Portugal	2016, 2019	Blueberry	Dieback, stem and twig blight	*N. rosae, N. scalabiensis, N. vaccinii and N. vacciniicola*	(Santos et al., 2022)
South Africa	2023	Blueberry	Leaf and twig blight	*N. rosae, N. hispanica*, and *N. longiappendiculata*	(Van der Vyver et al., 2025)
Spain	2015	Blueberry	Canker and twig dieback	*N. clavispora (pre. Pestalotiopsis clavispora)*	(Borrero et al., 2018)
Spain	2013/14	Strawberry	Root and crown rot	*Pestalotiopsis clavispora (N. clavispora)*	(Chamorro et al., 2016)
Taiwan	2018	Strawberry	Leaf blight and crown rot	*N. rosae*	(Wu et al., 2021)
Taiwan	2019	Jabuticaba	Leaf brown blight	*N. formicarum*	(Lin et al., 2022)
Thailand	2019	Mangrove trees	Endophyte form	*Neopestalotiopsis alpapicalis* sp. nov	(Kumar et al., 2019)
Thailand	2019	Rubber tree	Leaf spot and leaf fall	*N. cubana* and *N. formicarum*	(Pornsuriya et al., 2020)
Uruguay	2016	Strawberry	Root and crown rot	*N. clavispora*	(MacHín et al., 2019)
USA (Alabama)	2022	Strawberry	Leaf spot and fruit rot	*Neopestalotiopsis* spp.	(Conner, 2022)
USA (Arkansas)	2021	Strawberry	Leaf spot and fruit rot	*Neopestalotiopsis* spp.	(Cato, 2024)
USA (California)	2021	Strawberry	Root and crown rot	*N. rosae*	(Lawrence et al., 2023)
USA (Connecticut)	2023	Strawberry	Leaf spot and petiole blight	*Neopestalotiopsis* spp.	(Salvas et al., 2024)
USA (Delaware)	2023	Strawberry	Leaf spot, crown and fruit rot	*N. vaccinii*	(Gangwar et al., 2025)
USA (Florida)	1972	Strawberry	Fruit rot	*N. rosae*	(Howard and Albregts, 1973)
USA (Florida)	2017	Strawberry	Fruit rot	*N. rosae*	(Baggio et al., 2021)
USA (Florida)	2019	Pomegranate	Leaf and fruit spots	*N. rosae*	(Xavier et al., 2021)
USA (Georgia)	2021	Strawberry	Leaf spot and fruit rot	*Neopestalotiopsis* spp.	(Brannen, 2021)
USA (Georgia)	2023	Strawberry	Leaf spot and fruit Rot	New *Neopestalotiopsis* spp.	(Madrid et al., 2024)
USA (Georgia)	2021/22	Blueberry	Fruit rot	New *Neopestalotiopsis* spp.	(Beg and Oliver, 2025)
USA (Illinois)	2024	Strawberry	Leaf spot	*Neopestalotiopsis* spp.	(Wahle and Aly, 2024; Aly, 2024)
USA (Indiana)	2020	Strawberry	leaf spot	*Neopestalotiopsis* spp.	(Guan et al., 2023)
USA (Iowa)	2023	Blueberry	Leaf spot and dieback	*N. rosae*	(Dietsch et al., 2025)
USA (Kentucky)	2024	Strawberry	leaf, fruit, root, and crown infection	*Neopestalotiopsis* spp.	(Gauthier and Kaiser, 2025)
USA (Louisiana)	2022	Strawberry	Leaf spot and fruit rot	*Neopestalotiopsis* spp.	(Morgan, 2022)
USA (Missisippi)	2024	Strawberry	Fruit rot	*N. rosae*	(Madrid, 2024)
USA (Missouri)	2015	Grape	Leaf blight, fruit rot, trunk disease	*Neopestalotiopsis* spp.	(Volenberg, 2022)
USA (New Jersey)	2020	Strawberry	Leaf spot and fruit rot	*Neopestalotiopsis* spp.	(Wyenandt, 2020)
USA (North Carolina)	2022	Strawberry	Leaf, fruit, and crown rot	*Neopestalotiopsis* spp.	(Cline et al., 2024)
USA (Ohio)	2021	Strawberry	leaf spot and fruit rot	*Neopestalotiopsis* spp.	(Rotondo et al., 2023)
USA (Pennsylvania)	2020	Strawberry	Leaf spot	*Neopestalotiopsis* spp.	(Demchak, 2024)
USA (South Carolina)	2021	Strawberry	Leaf spot and fruit rot	*Neopestalotiopsis* spp.	(Kaur et al., 2023)
USA (Virginia)	2023	Strawberry	Leaf spot and crown rot	*N. rosae*	(Gangwar et al., 2025)
USA (Wisconsin)	2021	Strawberry	Crown and root infection	*Neopestalotiopsis* spp.	(Holland, 2022)

**Figure 4:**
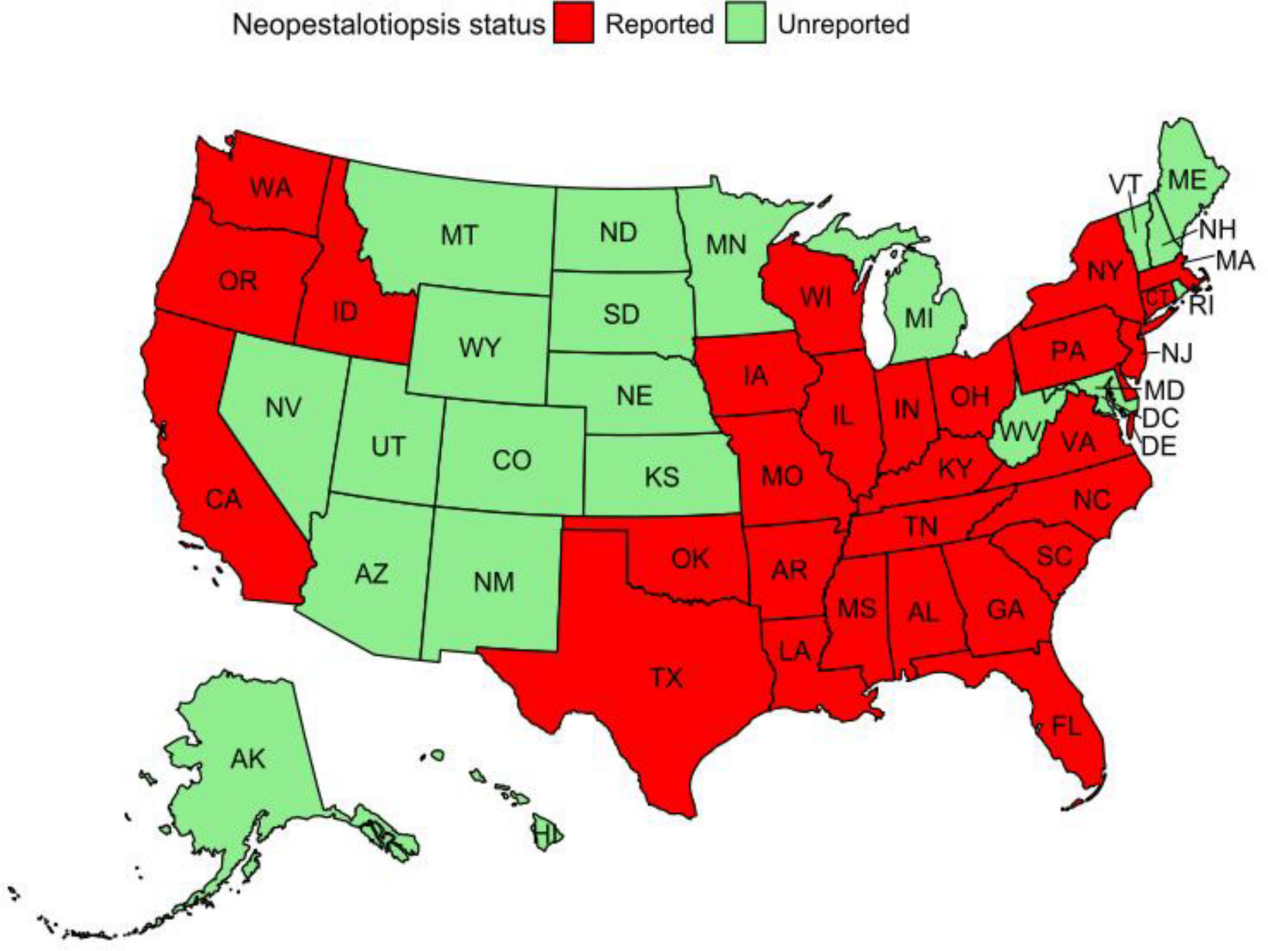
The published distribution of *Neopestalotiopsis* spp. across the U.S. is based on the data in **Table 1**. The figure does not show a specific location within each state.

Although this list is not exhaustive, *Neopestalotiopsis* is found in various regions worldwide (**Table 1**; **Figure 3**). In the United States, *Neopestalotiopsis rosae* (formerly *Pestalotiopsis longisetula* and *Pestalotia longisetula*) was first reported as the causal agent of strawberry fruit rot in Florida in 1972, leading to significant losses in both research plots and commercial fields (Baggio et al., 2021; Howard and Albregts, 1973). A major resurgence occurred in 2017, attributed to a new, attributed to a new lineage of *Neopestalotiopsis*. Since then, the pathogen has spread rapidly throughout the southeastern and Pacific regions of the United States (**Figure 4**, **Table 1**), including Georgia, South Carolina, North Carolina, Virginia, New Jersey, and Delaware (Baggio et al., 2021; Gangwar et al., 2025; Madrid et al., 2024; Miller et al., 2024).

*Neopestalotiopsis* infects nearly all parts of the strawberry plant, including roots, leaves, crowns, and fruits, and has a wide host range (**Table 1**). Its broad host range and aggressiveness under favorable environmental conditions underscore the significant threat that *Neopestalotiopsis* poses to global agricultural systems.

## Disease epidemiology, transmission dynamics, and host susceptibility

### Neopestalotiopsis symptoms

*Neopestalotiopsis* spp. has emerged as a globally significant pathogen in strawberry production, causing substantial yield losses due to various symptoms such as leaf lesions, root rot, crown rot, fruit rot, and plant death (Baggio et al., 2021; Beg and Oliver, 2025; Cline et al., 2024; Dardani et al., 2025; Uriel et al., 2025; Gilardi et al., 2019; Guan et al., 2023; McNally et al., 2023). Infected strawberry transplants typically exhibit leaf chlorosis, followed by wilting, stunted growth, and eventual death (Baggio et al., 2021; Rebollar-Alviter et al., 2020). Symptoms on the leaves initially appear as small, dark brown spots, approximately 1 mm in diameter, which expand concentrically and merge into larger necrotic areas, ultimately causing widespread leaf blight (Baggio et al., 2021; Rebollar-Alviter et al., 2020). Acervuli containing conidia are often present on these necrotic lesions, indicating active sporulation. On the petiole, dark brown, sunken lesions extend toward the crown, causing leaf wilting. Crown symptoms include irregular reddish discoloration of internal tissues with dark brown margins, while roots display dark brown coloration (Baggio et al., 2021; Rebollar-Alviter et al., 2020). On fruit, light tan to brown, slightly sunken, irregular lesions develop, gradually enlarging and becoming covered with numerous acervuli that contain shiny black droplets filled with conidia. Additionally, under humid conditions, dense white mycelium may form over these lesions (Baggio et al., 2021).

Symptoms associated with *Neopestalotiopsis* have also been observed in several other host crops (**Table 1**). Some reported symptoms include leaf blight, fruit rot, and trunk disease in grape (Kenfaoui et al., 2024; Volenberg, 2022); leaf spot, stem dieback, and fruit rot in blueberry and lingonberry (Dietsch et al., 2025; Novinscak et al., 2025); leaf and fruit spots in pomegranate (Xavier et al., 2021); and dry flower in macadamia (Prasannath et al., 2021).

### Epidemiology and transmission dynamics

A combination of conducive environmental factors, a virulent pathogen, and host susceptibility can trigger the pathogenic phase, leading to an epidemic. Outbreaks of *Neopestalotiopsis-*related diseases are particularly severe in warm, humid, and wet conditions (Baggio et al., 2021; Rebollar-Alviter et al., 2020). Optimal disease development occurs at temperatures of 25-30 °C, relative humidity above 80%, and prolonged leaf wetness exceeding 72 hours (Baggio et al., 2020**;**Belisário et al., 2020). The pathogen has been reported in major strawberry-producing regions (**Table 1**). Species and isolates collected from various times and locations exhibit varying levels of virulence on strawberry, complicating management strategies (Baggio et al., 2021; Rebollar-Alviter et al., 2020).

Although there is limited information on the germination and sporulation mechanisms of *Neopestalotiopsis* in strawberry, studies in Eucalyptus leaves and Macadamia flowers reported the production of germ tubes within one and a half hours and 12 hours post-inoculation (hpi), respectively (Belisário et al., 2020; Hariharan et al., 2025). In Macadamia flower, *N. macadamiae* formed appressoria, completed host tissue penetration and infection within 6 hpi, colonized within 24 hpi, and sporulated within 48 hpi (Hariharan et al., 2025).

The long-distance dissemination of *Neopestalotiopsis* is primarily attributed to the movement of infected nursery transplants (Baggio et al., 2021; McNally et al., 2023; Rebollar-Alviter et al., 2020). In the United States, the 2017 outbreak in Florida was caused by a novel, highly aggressive *Neopestalotiopsis* spp. traced to infected transplants obtained from nurseries in North Carolina (Baggio et al., 2021). By 2020, this outbreak had destroyed over 80 hectares of fields (Baggio et al., 2021). The same aggressive species was later detected in Canada, resulting in a 40% disease incidence in fruiting fields (McNally et al., 2023). Similarly, in Mexico, *N. rosae* has caused approximately 50% transplant losses in some epidemics, particularly in the cultivars Camino Real, Albion, and Festival, with initial outbreaks linked to open-field nurseries (Rebollar-Alviter et al., 2020).

Once the disease establishes in a commercial strawberry field, spores produced on infected tissues are mainly dispersed through infected runner plants, contaminated tools or implements, rain, and wind, initiating a secondary infection cycle (Baggio et al., 2021; Belisário et al., 2020; Maharachchikumbura et al., 2014; Rebollar-Alviter et al., 2020). Additionally, the pathogen can survive in plant debris and soil, potentially serving as a source of inoculum for subsequent growing seasons (Zuniga et al., 2024). Foliar disease symptoms are typically more severe in open-field cultivation, particularly in areas with frequent rainfall, which enhances splash dispersal of the pathogen. In contrast, high tunnel systems with protective plastic coverings reduce foliar disease by limiting leaf wetness; they do not prevent the introduction of the pathogen via infected transplants or the development of severe root and crown rot from soilborne inoculum (Acosta-González et al., 2024; Intriago-Reyna et al., 2021; Rebollar-Alviter et al., 2020).

### Host susceptibility

Variability in susceptibility among strawberry cultivars has been documented in both field and greenhouse settings. Several commercially grown cultivars in Florida, including Florida Beauty, Florida Radiance, Florida Brilliance, Sensation, Festival, and Winter Star, have shown significant susceptibility to this pathogen (Baggio et al., 2020). In a greenhouse trial evaluating cultivar susceptibility using both plug and bare-root transplants, Guan et al. (2023) found that widely cultivated varieties, such as Camino Real, Albion, San Andreas, Florida Brilliance, and Sweet Charlie, were highly susceptible to *Neopestalotiopsis*. In contrast, cultivars such as Galletta, AC Valley, Malwina, and Flavorfest consistently exhibited resistance regardless of the planting material used (Guan et al., 2023). A study on *N. rosae* inoculation on detached leaves in Mexico identified Marisol as the least susceptible cultivar, while Fortuna, Albion, Festival, and Fronteras were among the most susceptible (Uriel et al., 2025). Additionally, an exotic cultivar named Yasmin has been reported to exhibit moderate resistance (Alam et al., 2024). ​In North Carolina, some NC breeding lines and cultivars show good resistance to *Neopestalotiopsis* in preliminary screening (Heagy et al., 2025).

### *Neopestalotiopsis* pathogen detection

#### Morphoological identification to Serological method

Accurate and timely identification of plant pathogens is essential for effective disease management and preventing significant crop losses. Early and precise detection enables targeted control strategies that can reduce unnecessary pesticide applications and increase the likelihood of successful disease control.

### Morphological identification

Traditionally, *Neopestalotiopsis* has been identified based on disease symptoms and the colony and conidial morphology of cultures. However, symptom-based diagnosis can be challenging because similar symptoms may be caused by other pathogens, such as *Colletotrichum* spp., *Phytophthora cactorum*, and *Macrophomina phaseolina*, which also cause fruit rot, root rot, or leaf blight (Baggio et al., 2020, 2021). This overlap can lead to misdiagnoses during the early stages of the disease. While morphological identification, particularly through colony and conidial characteristics, can be helpful when isolating the pathogen from symptomatic tissues, it is insufficient for reliable species-level identification within the *Neopestalotiopsis* genus (Maharachchikumbura et al., 2014). This distinction is crucial given the variation in virulence among different *Neopestalotiopsis* isolates or species (Baggio et al., 2021), which is necessary for developing an appropriate management approach.

### Identification based on molecular phylogenetic-informed assays

To address the limitations of traditional fungal identification methods, molecular techniques have become increasingly important. Maharachchikumbura et al. (2014) used sequence data from the internal transcribed spacer (ITS) region, partial β-tubulin (tub) gene, and translation elongation factor 1-alpha (tef1-α) gene, along with conidial morphology, to differentiate *Pestalotiopsis*-like fungi into three genera: *Pestalotiopsis*, *Neopestalotiopsis*, and *Pseudopestalotiopsi*s. These genetic markers have been applied in phylogenetic analyses using whole-genome or amplicon sequencing to identify *Neopestalotiopsis* species and investigate their evolutionary relationships with known species (Dardani et al., 2025; Uriel et al., 2025; Han et al., 2024; Heng et al., 2025; Maharachchikumbura et al., 2014; Rebello et al., 2023).

### PCR assays for specific detection

Next-generation sequencing (NGS) platforms play a crucial role in comprehensive genome analysis, enabling the detection of novel and emerging fungal pathogens, with or without prior sequence knowledge. Using sequence data, researchers have designed genetic markers to facilitate the rapid, accurate differentiation of closely related *Neopestalotiopsis* spp. isolated from symptomatic strawberry tissues via molecular diagnostics (Baggio et al., 2021; Kaur et al., 2023; Rebello et al., 2023). Kaur et al. (2023) developed a PCR-RFLP assay targeting the β-tubulin gene with Bt2a/Bt2b primers, followed by *Bsa*WI digestion. This method produced two distinct fragments (290 bp and 130 bp) for the newly identified aggressive *Neopestalotiopsis* spp., whereas known *N. rosae* and other circumscribed species yielded a single 420 bp fragment. The difference in restriction digestion resulted from a species-specific single-nucleotide polymorphism (SNP) in the β-tubulin gene, which created a *Bsa*WI restriction site present only in the new aggressive isolates (Baggio et al., 2021; Kaur et al., 2023).

While effective in distinguishing the new aggressive species from previously classified *N. rosae* and related species, this method has limitations in differentiating among all *Neopestalotiopsis* spp (Baggio et al., 2021; Kaur et al., 2023). Similarly, Rebello et al. (2023) developed a high-resolution melting (HRM) assay using two primer sets (Neo_Tub2_A1F/Neo_Tub2_A1R and Neo_Tub2_B1F/Neo_Tub2_B1R) that target polymorphic regions of the β-tubulin gene. This method successfully differentiated the newly identified aggressive *Neopestalotiopsis* spp. from previously classified *N. rosae* and other species based on distinct melting profiles. However, this HRM assay is recommended only for *Neopestalotiopsis* isolates from strawberries, given that the genus *Neopestalotiopsis* is taxonomically complex and its species are considered polyphyletic (Rebello et al., 2023). A critical caveat for these assays is that they are designed for and are most reliable with strawberry isolates; their accuracy may be compromised when applied to the broader, taxonomically complex genus found on other hosts (Rebello et al., 2023). Future development of broader and more specific assays will be essential to enhance diagnostic accuracy, enable precise species-level identification, and expand applicability across various hosts.

### Isothermal amplification and field-deployable diagnostics

Despite significant advances in molecular diagnostics, current molecular techniques are primarily performed in laboratory settings. This limits their use in field settings due to factors such as cost, time requirements, complexity, and the need for sophisticated equipment. For in-field diagnostics, isothermal amplification techniques such as Loop-Mediated Isothermal Amplification (LAMP) (Notomi et al., 2000) and Recombinase Polymerase Amplification (RPA) (Piepenburg et al., 2006) offer a promising alternative. These methods are rapid, cost-effective, require minimal instrumentation, and can be applied to crude plant extracts. However, their success hinges on the development of species-specific genomic markers. They have been successfully deployed for other major strawberry pathogens like *Phytophthora* (Lu et al., 2021; Munawar et al., 2020; Siegieda et al., 2021), *Colletotrichum* (Wu et al., 2019; Zhang et al., 2016), *Botrytis* (Chen et al., 2022; Hu et al., 2017; Vielba-Fernández et al., 2023), and *Fusarium* (Hu et al., 2023), but their development for *Neopestalotiopsis* awaits species-specific marker discovery and validation. Therefore, designing and validating novel species- or pathotype-specific markers is essential for accurate identification of *Neopestalotiopsis* spp. The future of field-based diagnostics likely lies in the integration of these technologies. CRISPR-based platforms coupled with RPA/LAMP offer unprecedented specificity and sensitivity for on-site detection, as demonstrated for other fungi (Dong et al., 2024; Gong et al., 2025; Tian et al., 2025; Wang et al., 2025a, 2023; Zheng et al., 2025). For instance, Tian et al. (2025) developed a one-pot LAMP-CRISPR/Cas12b assay for identifying *Fusarium oxysporum* and *Colletotrichum siamense* in strawberries. This assay demonstrated a sensitivity of 10 DNA copies within 30 minutes and required no specialized equipment, making it particularly suitable for on-site early disease detection (Tian et al., 2025). Given these promising advancements in ease of use and speed, similar CRISPR-based platforms, isothermal amplification techniques, and biosensors could potentially be adapted for the rapid detection of *Neopestalotiopsis* spp. in the future. Furthermore, non-invasive technologies, such as hyperspectral imaging powered by machine learning, can detect physiological changes in plants before symptom onset (Castro-Valdecantos et al., 2024; Chun et al., 2024; Ou et al., 2024; Szechyńska-Hebda et al., 2025). For ultimate portability, microfluidic “lab-on-a-chip” systems that integrate sample preparation, amplification, and detection into a single, automated platform represent a frontier in rapid phytopathogen diagnostics (Guo et al., 2021; Zhao et al., 2024). These compact, automated systems facilitate high-throughput, on-field diagnostics with minimal manual handling and technical skills, offering a robust, scalable solution for rapid phytopathogen detection and proactive disease management (Paul et al., 2021; Shymanovich et al., 2024; Yadav and Yadav, 2025; Zhang et al., 2023).

### Serological methods

Serological assays based on the antibody-antigen principle may be employed as a cost-effective and straightforward diagnostic alternative for *Neopestalotiopsis* in the future. However, they exhibit low sensitivity, particularly for detecting low pathogen loads or latent infections (Luchi et al., 2020). Their diagnostic reliability can be improved by integrating them with molecular confirmatory methods, thereby enhancing diagnostic accuracy.

## *Neopestalotiopsis* genomics

The rapid advancement of next-generation sequencing (NGS) technologies has revolutionized genomic and genetic research on fungal pathogens. These technologies have enabled the development of innovative tools and methodologies that significantly improve the accuracy and efficiency of pathogen identification, species differentiation, and disease diagnosis. Beyond diagnostics, NGS has become indispensable for studying fungal population genetics, taxonomy, and evolutionary biology, providing deeper insights into genetic diversity, speciation, and phylogenetic relationships (Naqvi et al., 2025). However, genomic and genetic studies of *Neopestalotiopsis* have lagged due to recent reclassifications, taxonomic complexity, and the historical paucity of focused research.

As of September 29, 2025, the National Center for Biotechnology Information (NCBI) genome database listed six publicly available *Neopestalotiopsis* genomes isolated from various plant hosts. Of these, only three were isolated specifically from strawberry tissues (Han et al., 2024; Hsu et al., 2022). The first high-quality genome assembly of *Neopestalotiopsis* was presented by Hsu et al. (2022), based on the *N. rosae* strain ML1664, which was isolated from the crown tissue of a diseased strawberry plant in Hsinchu County, Taiwan (Wu et al., 2021). This assembled genome comprised 18 contigs totaling 53.78 Mb, and achieved 98.4% completeness, as evaluated by Benchmarking Universal Single-Copy Orthologs (BUSCO) analysis. From the genome of strain ML1664, a total of 15,966 putative protein-coding genes, 2,234 candidate secreted peptides, and 76 biosynthesis gene clusters were predicted, hinting at a complex repertoire of potential virulence factors (Hsu et al., 2022).

Recently, Han et al. (2024) generated two chromosome-scale reference genome assemblies from isolates with varying virulence levels: *Neopestalotiopsis* spp. 19-02 (highly virulent) and *N. rosae* 13-481 (moderately virulent). The genome sizes were 52.6 Mb for 19–02 and 50.1 Mb for 13-481, with both assemblies showing 98.5% completeness of conserved core genes, as determined by BUSCO analysis. In total, 15,517 genes were predicted in the 19–02 assembly, while 15,022 genes were expected in the 13–481 assembly (Han et al., 2024). Significantly, this study confirmed that the *Neopestalotiopsis* genome contains seven basic chromosomes (Han et al., 2024). All three genome assemblies were developed using a combination of Oxford Nanopore and Illumina sequencing platforms, yielding high-quality, comprehensive representations of the *Neopestalotiopsis* genomes (Han et al., 2024; Hsu et al., 2022).

Comparative genomic analysis revealed a high degree of collinearity between the 19–02 and 13–481 genomes (Han et al., 2024). Structural variants were distributed across all seven chromosomes, with the highest number observed on chromosome 1, suggesting potential differences in the evolutionary origins of these isolates (Han et al., 2024). Furthermore, both assemblies demonstrated strong synteny with the ML1664 genome. Out of the 18 contigs in the ML1664 assembly, 14 aligned with the chromosome-scale assemblies of the 19–02 and 13–481 genomes (Han et al., 2024; Hsu et al., 2022).

While existing genome assemblies of *Neopestalotiopsis* spp. have provided a foundation for future genomic and molecular studies, a more comprehensive understanding of their virulence, pathogenicity, and host specificity is still lacking. Key genomic factors, including genome structure, gene duplication, nucleotide variation, transposable element-mediated gene neofunctionalization, horizontal gene transfer, accessory or mobile chromosomes, secondary metabolites, and the evolution of pathogenicity-related genes, are known to contribute to the diversification and specialization of fungal pathogens (Francis et al., 2023; Rokas et al., 2020; Sauters and Rokas, 2025; Witte et al, 2021). However, these factors remain largely unexplored in *Neopestalotiopsis* spp. In *N. rosae* strain ML1664, it has been predicted that biosynthesis gene clusters contain a diverse array of compounds, including beta-lactone, ribosomally synthesized and post-translationally modified peptides (RiPPs), indoles, terpenes, type I polyketide synthases (T1PKSs), type III polyketide synthases (T3PKS), nonribosomal peptide synthetases (NRPSs), NRPS-like sequences, and hybrids (Hsu et al., 2022). These compounds may contribute to the pathogenicity of *Neopestalotiopsis*; however, further functional validation is needed to elucidate how they facilitate infection and exploit host resources. NGS technologies present a valuable opportunity to investigate these genomic factors in detail. This can lead to deeper insights into the molecular mechanisms underlying *Neopestalotiopsis* pathogenicity, virulence evolution, and host adaptation. Future studies that integrate comparative and functional genomics could not only reveal the molecular mechanisms of infection and the evolutionary dynamics of this emerging genus but also aid in developing robust tools for accurate taxonomic clarification, pathogen identification, early detection, species-level diagnostics, and resistance breeding strategies in host plants.

### Current disease management strategies

#### Cultural control to Novel sources of disease resistance

Effective management of *Neopestalotiopsis* infections requires an integrated approach that addresses multiple factors influencing disease development and spread. However, current information on a comprehensive management strategy for *Neopestalotiopsis* is limited. Key factors to consider include environmental conditions, access to disease-free planting material, early and accurate diagnostics, field sanitation, the availability of effective chemical and biological control strategies, and the identification of novel sources of disease resistance (Acosta-González et al., 2024; Alam et al., 2024; Amrutha and Vijayaraghavan, 2018; Kaur et al., 2023; Rebollar-Alviter et al., 2020).

### Cultural control

Initial outbreaks of *Neopestalotiopsis* have often been linked to infected nursery transplants, highlighting the critical importance of using certified, disease-free propagation materials (Baggio et al., 2021; McNally et al., 2023; Rebollar-Alviter et al., 2020). Pathogens can also spread via farm tools; therefore, it is essential to clean and disinfect these tools after each use. Moreover, the pathogen is known to survive in soil and plant debris (Zuniga et al., 2024), underscoring the importance of rigorous practices to reduce inoculum levels in the soil and prevent reinfection in subsequent cropping cycles. Strategies such as the immediate removal or burial of infected plant residues after harvest can significantly limit the pathogen’s survival and spread. Additionally, crop rotation with non-host species could help break the disease cycle and suppress pathogen persistence in the soil and field. This method has been effective for other fungal diseases in strawberries (Shrestha et al., 2024).

### Chemical control

Soil fumigation is also an effective soil disinfestation measure for managing soilborne fungal pathogens. Pre-plant fumigation with formulations of 1,3-dichloropropene and chloropicrin (e.g., Pic-Clor 60, Pic-Clor 80, or Telone C-35) or metam potassium is effective in reducing *Neopestalotiopsis* inoculum in both soil and strawberry crowns (Alonzo et al., 2025; Zuniga et al., 2024).

Fungicides have long been recognized as an effective approach for managing fungal diseases in crops; however, few studies have examined their use to manage *Neopestalotiopsis* in strawberries. In an *in vitro* assay, several compounds demonstrated 100% inhibition of *N. clavispora*. These included carbendazim 12% + mancozeb 63% (Saaf), cymoxanil 8% + mancozeb 64% (Curzate M8), copper hydroxide 77WP (Kocide), copper oxychloride 50WP (Fytolan), propineb 70WP (Antracol), and the Bordeaux Mixture (Amrutha and Vijayaraghavan, 2018). Under field conditions, both propineb 70WP (Antracol) and carbendazim 12% + mancozeb 63% (Saaf) demonstrated high efficacy, resulting in more than 74% disease reduction (Amrutha and Vijayaraghavan, 2018).

In Egypt, an *in vitro* evaluation of the fungicides thiram (1000 ppm) and hymexazole (1250 ppm) demonstrated that they inhibited the mycelial growth of *N. rosae* by more than 90% (Essa et al., 2018). Under greenhouse conditions, thiram and hymexazole reduced the severity of crown and root rot, achieving efficacy rates of 80% and 76%, respectively, on the cv. Fortuna, and 82% and 79% on the cv. Festival (Essa et al., 2018). In a large-scale study conducted in Florida, Baggio et al. (2023) evaluated 30 commercially available fungicides *in vitro* and identified several effective treatments for managing *N. rosae*. The most effective fungicides included single-site fungicides such as fludioxonil and fluazinam, as well as sterol demethylation inhibitors, and multisite fungicides such as captan, thiram, and chlorothalonil (Baggio et al., 2023). Furthermore, alternating cyprodinil + fludioxonil with thiram was found to be the most reliable management strategy for controlling *Neopestalotiopsis* fruit rot under field conditions (Baggio et al., 2023). In contrast, fungicides from FRAC groups 1 (methyl benzimidazole carbamates) and 7 (succinate dehydrogenase inhibitors) were ineffective (Baggio et al., 2023). A recent study by Acosta-González et al. (2024) in Mexico reported high efficacy for several treatments, including prochloraz, prochloraz combined with thiram, cyprodinil combined with fludioxonil, and a root-dip treatment using pydiflumetofen plus fludioxonil. The study found that preventive root-dip applications before transplanting were more effective than post-transplant treatments. Based on these findings, the researchers recommended a two-step strategy for optimal disease suppression: an initial root dip before transplanting, followed by a crown drench 8 to 10 days later (Acosta-González et al., 2024). Moreover, regulatory and resistance-related challenges associated with the use of certain fungicides must be considered when developing management strategies. For instance, fluazinam and chlorothalonil are not recommended for use in fruit production fields due to potential food-residue concerns (Baggio et al., 2023). The fungicide carbendazim, which is effective against *N. clavispora* in India (Amrutha and Vijayaraghavan, 2018), is currently restricted in the United States because of regulatory concerns (Baggio et al., 2023). In Thailand, the use of this treatment is advised with caution due to confirmed resistance observed in *Pestalotiopsis* spp. isolates to benzimidazoles, which is associated with mutations in the β-tubulin gene (Kummanid et al., 2017). Similarly, quinone outside inhibitor (QoI) fungicides, particularly those in FRAC Group 11, such as azoxystrobin, have been discouraged for managing *Neopestalotiopsis* spp. in Florida due to resistance associated with a specific mutation in the cytochrome b gene (Baggio et al., 2023; Hu et al., 2017) and the critical need to optimize use of these products to target *Colletotrichum* pathogens. Nevertheless, regional variability in isolate sensitivity to fungicides has also been noted. For example, azoxystrobin was reported to significantly inhibit the mycelial growth of *N. rosae*, with inhibition rates of 79.4% in strawberry crowns and 85% in leaves in Mexico (Kummanid et al., 2017). Another study in Thailand demonstrated 100% inhibition of the mycelial growth of *Neopestalotiopsis* and *Pseudopestalotiopsis* isolates by a combination of azoxystrobin, tebucanozole, and prochloraz (Darapanit et al., 2021). These contrasting findings suggest that fungicide efficacy may vary based on geographic origin, environmental conditions, and the genetic diversity of *Neopestalotiopsis* isolates. This highlights the importance of localized resistance monitoring and fungicide sensitivity profiling in developing effective management strategies.

### Biological control and botanical pesticides

Biological control and botanical pesticides offer promising, non-toxic alternatives for managing diseases caused by *Neopestalotiopsis* in crop plants. *In vitro* evaluations have shown that specific microbial antagonists can effectively inhibit the growth of *N. clavispora*. For example, *Trichoderma asperellum* and *Pseudomonas fluorescens* reduced fungal growth by 66.7% and 56.7%, respectively (Amrutha and Vijayaraghavan, 2018). Under field conditions, the foliar application of *T. asperellum* resulted in a 75.8% reduction in disease incidence compared to the untreated control, making it the second most effective treatment after the chemical fungicide propineb 70WP (Antracol) (Amrutha and Vijayaraghavan, 2018). Additionally, *T. asperellum* has been reported to suppress disease symptoms caused by *N. rosae*, underscoring its potential as a key component of integrated disease management programs, either when used alone or in combination with chemical fungicides (Acosta-González et al., 2024). In addition to fungal biocontrol agents, bacterial antagonists like *Bacillus cereus* (strain Bce-2) have demonstrated significant effectiveness against *N. clavispora* by triggering biochemical and molecular defense responses in host plants (Zhang et al., 2024). *Bacillus cereus* Bce-2 showed an *in vitro* inhibition rate of 79.48% (Zhang et al., 2024). Field studies further indicated that applying a *Bacillus cereus* fermentation solution (BCFS) as a pre-treatment for strawberry plants reduced the disease index by 57.85% (Zhang et al., 2024). The use of BCFS also increased the activity of defense-related enzymes, including peroxidase (POD), superoxide dismutase (SOD), catalase (CAT), and phenylalanine ammonia-lyase (PAL). Furthermore, it enhanced the expression of key defense-related genes, including WRKY22, WRKY29, ChiB, RbohD, HSP90, Rd19, PR1, and PP2C, in the 512 leaves of strawberry plants (Zhang et al., 2024).

Crude plant extracts have been investigated as potential biofungicides or biocontrol agents for managing *Neopestalotiopsis* spp. For instance, Darapanit et al. (2021) reported that oil extracts from clove, ginger, lemongrass, roselle, and turmeric effectively inhibited the growth of *Neopestalotiopsis* and *Pseudopestalotiopsis*. Notably, extracts of clove and turmeric, when used at a concentration of 10000 mg/L, completely inhibited the *in vitro* development of these pathogens. Additionally, cinnamon oil-encapsulated lipid nanoemulsions (CiLN) demonstrated significant antifungal activity against *N. rosae*, achieving 100% effectiveness in preventing and 90.42% effectiveness in treating strawberry diseases, without adversely affecting plant growth (Tran et al., 2023). These studies demonstrate that microbial antagonists, such as *T. asperellum* and *B. cereus*, and biofungicides are promising, eco-friendly tools for managing diseases caused by *Neopestalotiopsis*, thereby supporting their integration into sustainable plant protection strategies.

### Novel sources of disease resistance

The development and use of resistant cultivars represent the most sustainable approach to disease management. Some documented cultivars exhibit resistance or reduced susceptibility (Baggio et al., 2020; Guan et al., 2023; Uriel et al., 2025). Additionally, a comprehensive evaluation by the University of Florida’s strawberry breeding program assessed 1,578 breeding lines and discovered that approximately 12% of elite University of Florida (UF) germplasm exhibited resistance to the pathogen (Alam et al., 2024). Notably, this resistance was inadvertently introduced into the UF germplasm via the exotic cultivar Yasmin, a powerful source of resistance and a valuable genetic resource for future breeding efforts (Alam et al., 2024). Despite the global spread of disease and increasing crop losses, no effective resistance has been identified among the widely grown commercial cultivars. This underscores the urgent need for improved disease-control measures and breeding programs to develop more resilient cultivars.

## Innovative molecular breeding and genomics techniques for enhancing host plant resistance to *Neopestalotiopsis* spp.

**Figure 5:**
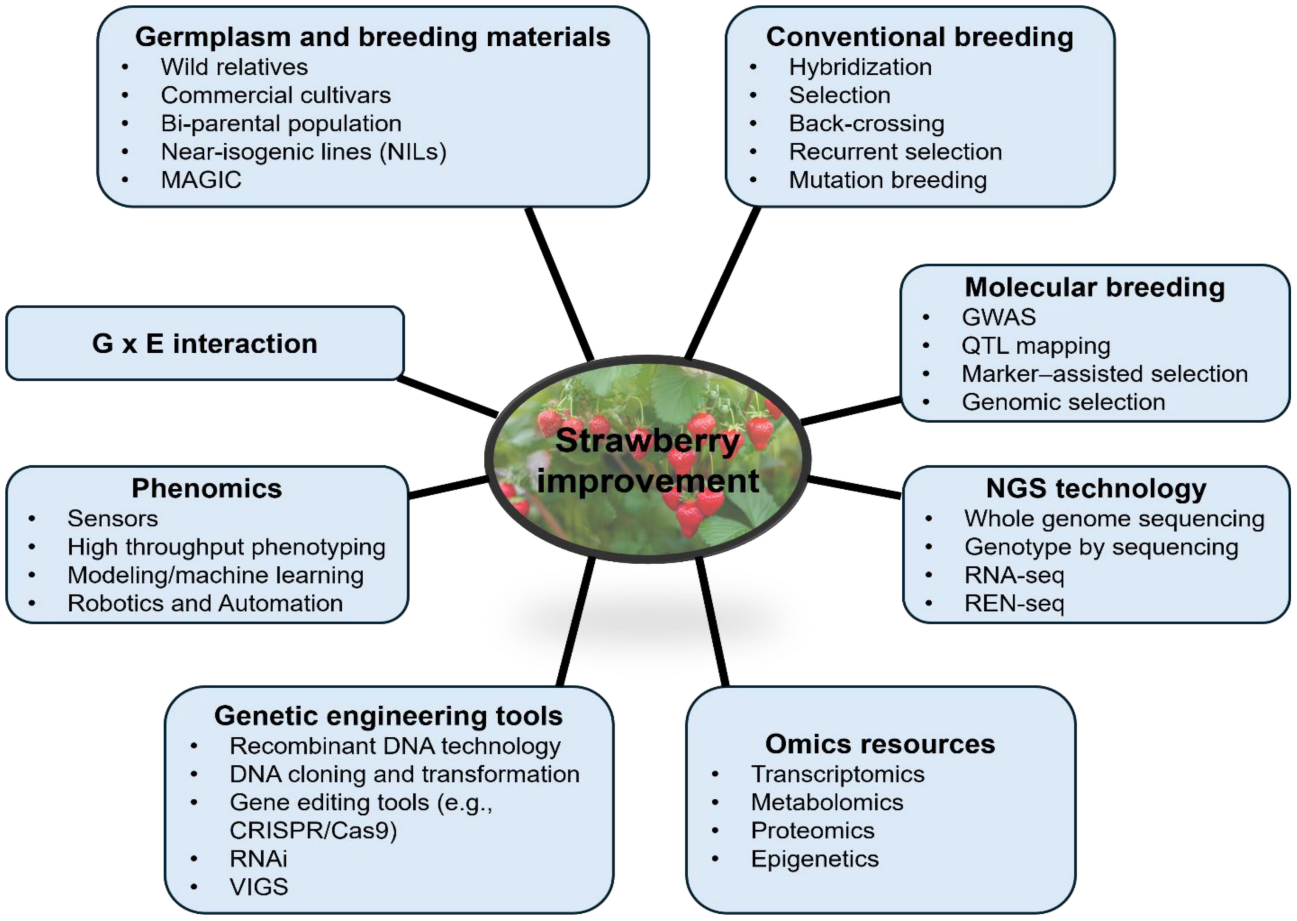
Strawberry breeding resources and methods for improvement.

Recent advances in strawberry genomics have significantly enhanced the potential for molecular tools in breeding programs. The development of a high-density SNP genotyping array, known as FanaSNP (Hardigan et al., 2020), ‘Camarosa’ (Edger et al., 2019), and the haplotype-phased genomes of the cultivar ‘Royal Royce’ (Hardigan et al., 2021) together provide a comprehensive genomic framework for strawberry breeding. These genomic resources, combined with NGS technologies, have enabled the identification of resistance (*R*) genes linked to disease resistance in octoploid strawberries (Barbey et al., 2019; Edger et al., 2019). Furthermore, Barbey et al. (2019) developed resistance gene enrichment sequencing (RenSeq) probe panels, which are essential for targeted sequencing of R genes and accelerating the incorporation of disease-resistance traits into strawberry breeding programs. The understanding of the genetic basis for disease resistance against *Neopestalotiopsis* spp. remains limited, primarily due to the lack of comprehensive genomic information available to researchers. Historically, pestalotioid fungi have been regarded as secondary or opportunistic pathogens of strawberries, resulting in minimal research attention (Baggio et al., 2020, 2021). However, the recent global outbreaks of virulent strains has prompted increased interest in developing resistant cultivars and understanding the underlying mechanisms of resistance to *Neopestalotiopsis* spp. (**Figure 5**).

### Genome-wide association study

A genome-wide association study (GWAS) conducted by Alam et al. (2024) examined the genetic basis of resistance to *Neopestalotiopsis* spp. using Florida strawberry germplasm and wild relatives, alongside the Axiom 50K FanaSNP array (Hardigan et al., 2021) and the Royal Royce reference genome (Hardigan et al., 2021). This study is currently the only one focused on this specific area. The researchers identified several candidate genes related to pattern recognition receptors, intracellular nucleotide-binding leucine-rich repeats, and downstream components of plant defense pathways. These genes co-localized with the resistance loci *RNp1* and *RNp2*, located on chromosomes 6B and 7A, respectively (Alam et al., 2024). The *RNp1* locus was commonly found in UF germplasm, but its effect was relatively small and insufficient on its own to confer strong resistance. In contrast, the rarer *RNp2* locus demonstrated a stronger association with resistance and was introduced via the Mediterranean cultivar Yasmin. However, Yasmin itself exhibited only marginal resistance, likely due to other genetic interactions. Despite this limitation, *RNp2* enhanced resistance in 42% of the offspring resulting from a cross between Yasmin and susceptible UF selections, making it a promising candidate for marker-assisted selection (MAS) (Alam et al., 2024). At the same time, this study highlights potential quantitative trait loci (QTL) and candidate genes for MAS concerning *Neopestalotiopsis* spp. Further research is needed to validate the functional roles of the causal genes and markers linked to the RNp1 and RNp2 loci, addressing resistance. Additionally, Alam et al. (2024) mentioned that their study relied on only three different isolates recovered from symptomatic strawberry leaves in Florida. Consequently, the effectiveness of these loci and associated genes against global *Neopestalotiopsis* isolates remains to be validated to ensure their broad applicability for MAS and the durability of resistant cultivars.

### RNA sequencing

Identifying candidate genes and incorporating them into elite breeding lines is crucial for developing new cultivars with durable resistance to the pathogen. High-throughput technologies, such as RNA sequencing (RNA-seq) and gene regulatory analysis, have become powerful tools for understanding host defense mechanisms and discovering resistance-associated genes involved in host-pathogen interactions (Adhikari et al., 2022; Wang et al., 2025b; Xiong et al., 2018). The integration of expression quantitative trait loci (eQTL) analysis with GWAS has also proven effective in linking gene-expression variability to genetic variation, thereby enhancing candidate-gene discovery (Barbey et al., 2019; Fan et al., 2022). For example, the combined use of eQTL and GWAS has successfully identified key genes controlling fruit flavor in strawberries (Fan et al., 2022), demonstrating the effectiveness of this integrative approach. Leveraging multi-omics data, including traditional QTL mapping, GWAS, eQTL mapping, RNA-seq, metabolomics, and proteomics, can significantly narrow down QTL confidence intervals and improve resolution for reliably identifying candidate genes underlying complex traits like disease resistance (Li et al., 2024; Varadharajan et al., 2025; Wang et al., 2025b; Zou et al., 2025). In future studies, these integrated strategies can provide a comprehensive framework for accelerating the development of resistant cultivars against *Neopestalotiopsis* spp. through molecular breeding.

### Genomic selection

Genomic selection (GS) represents a powerful alternative to MAS for improving complex polygenic traits, such as disease resistance (Kumari et al., 2024). Unlike MAS, which typically focuses on a limited number of major QTLs or markers, GS considers both major and minor markers across the entire genome. This broader approach enhances prediction accuracy, accelerates genetic gains, and improves the efficiency of selection for complex traits (Kumari et al., 2024). Alam et al. (2024) evaluated the predictive capabilities of two GS models: the classical genomic best linear unbiased prediction (GBLUP) and GBLUP augmented with the top three GWAS markers as fixed effects. Their findings showed predictive abilities ranging from 0.33 to 0.59, indicating the potential of GS for improving resistance to *Neopestalotiopsis* species (Alam et al., 2024). However, incorporating GWAS-derived markers as fixed effects did not improve predictive performance (Alam et al., 2024). This suggests that the selected GWAS markers may not be causative and may display different patterns of linkage disequilibrium or allele frequencies between the training and testing populations (Alam et al., 2024; Witte, 2010). Consequently, this weakens the marker-trait associations and diminishes the predictive power of GS when using those specific markers. These results underscore the necessity for future studies to identify and validate genuine causal variants or functional markers. Approaches such as fine mapping, functional genomics, multi-omics, colocalization analyses, and Mendelian randomization can be employed to achieve this (Adebiyi et al., 2021; King et al., 2021; Witte, 2010). Incorporating validated causal variants as fixed effects in GS models has significant potential to enhance predictive accuracy and improve the reliability of genomic predictions (Park et al., 2025). In future research, the identified genetic markers and candidate genes (Alam et al., 2024) can serve as valuable starting points for reverse genetics approaches to enhance strawberry resistance to *Neopestalotiopsis* spp.

### Gene editing technologies

New targeted gene editing technologies, such as CRISPR/Cas9, as well as chemical or physical mutagenesis, can be utilized to examine the functional roles of specific allelic variants or genes (Härtl et al., 2017; Park et al., 2025; Sharma et al., 2022; Xing et al., 2018). These methods enable researchers to establish causal relationships between gene function and resistance phenotypes, facilitating the identification of key genes involved in pathogen defense. This knowledge can inform breeding strategies by prioritizing the incorporation of resistance genes into new cultivars, ultimately leading to the development of strawberry varieties that are resistant to *Neopestalotiopsis* spp.

CRISPR/Cas9 is an advanced genome-editing technology that has become an invaluable tool in agriculture and plant research (Tuncel et al., 2025). It allows for precise, targeted DNA modifications, facilitating functional genomics studies, accelerating crop improvement, and introducing novel genetic variants (Li et al., 2017; Lu and Zhu, 2017; Shimatani et al., 2017). A key component of CRISPR/Cas9-mediated gene editing is the single-guide RNA (sgRNA), which guides the Cas9 enzyme to the target DNA sequence while minimizing off-target effects. Several online tools exist to assist with sgRNA design, including CHOPCHOP (Labun et al., 2019), CRISPOR (Concordet and Haeussler, 2018), CRISPR direct (Naito et al., 2015), CRISPR-P (Lei et al., 2014), and CRISPR RGEN Tools (Bae et al., 2014). These tools help researchers optimize sgRNA selection based on efficiency and specificity (Bae et al., 2014; Labun et al., 2019; Lei et al., 2014; Naito et al., 2015). The CRISPR/Cas9 system has been successfully applied in strawberries (*Fragaria* × *ananassa*) for targeted mutagenesis (Hernández-Amasifuen et al., 2024; Martín-Pizarro et al., 2019; Wilson et al., 2019; Xing et al., 2018). For example, Hernández-Amasifuen et al. (2024) designed two sgRNAs using CHOPCHOP to target the *FaRALF33* gene. This gene-editing strategy aimed to reduce susceptibility to *Colletotrichum acutatum*, a significant fungal pathogen affecting strawberries (Hernández-Amasifuen et al., 2024). This example underscores the potential of CRISPR/Cas9 to enhance disease resistance through precise genome modification in strawberries.

### RNA interference

RNA interference (RNAi) is a powerful and precise gene-regulatory mechanism that utilizes a natural antiviral defense mechanism found in eukaryotes (Mezzetti et al., 2020; Obbard et al., 2009). Its use in plant biotechnology has shown considerable promise for genetic improvement and disease management (Kuo and Falk, 2020; Luca et al., 2024; Mezzetti et al., 2020). In strawberries, RNAi has been effectively employed to modify endogenous genes and manage diseases (Härtl et al., 2017; Kadomura-Ishikawa et al., 2015; Lin et al., 2013; Luca et al., 2024). For example, Luca et al. (2024) effectively controlled gray mold disease in cultivated strawberries by silencing *Dicer-like 1* (*DCL1*) and *2* (*DCL2*) genes of *Botrytis cinerea* using RNAi-based strategies. Consequently, future RNAi-based strategies offer promising opportunities to validate candidate resistance genes and to develop effective measures to control *Neopestalotiopsis* through resistance breeding.

## Conclusions

The emergence of *Neopestalotiopsis* poses a significant threat to strawberry cultivation, prompting increased attention from researchers and stakeholders. Progress has been made in disease management strategies, pathogen detection tools, and initial resistance breeding efforts, but challenges remain. Taxonomic complexity, lack of resistant cultivars, and limited understanding of the host’s defense mechanisms hinder effective control. To develop sustainable disease management strategies, an integrated and multidisciplinary approach is essential. Advances in genomics for both strawberry and *Neopestalotiopsis* provide opportunities to understand host-pathogen interactions, accelerate resistance breeding, and design targeted management strategies. Collaborative research will be imperative to ensuring productive and disease-resilient strawberry cultivation in light of this emerging pathogen.
